# Additive Manufacturing of Micromanipulator Mounted on a Glass Capillary for Biological Applications

**DOI:** 10.3390/mi11020174

**Published:** 2020-02-07

**Authors:** Shingo Kozaki, Yukihito Moritoki, Taichi Furukawa, Hikaru Akieda, Tatsuto Kageyama, Junji Fukuda, Shoji Maruo

**Affiliations:** 1 Graduate School of Engineering Science, Yokohama National University, 79-5 Tokiwadai, Hodogaya, Yokohama 240-8501, Japan; kozaki-shingo-yh@ynu.jp (S.K.); akieda-hikaru-dh@ynu.jp (H.A.); 2 College of Engineering Science, Yokohama National University, 79-5 Tokiwadai, Hodogaya, Yokohama 240-8501, Japan; moritoki-yukihito-zm@ynu.jp; 3 Faculty of Engineering, Yokohama National University, 79-5 Tokiwadai, Hodogaya, Yokohama 240-8501, Japan; furukawa-taichi-xp@ynu.ac.jp (T.F.); kageyama-tatsuto-tp@ynu.ac.jp (T.K.); fukuda@ynu.ac.jp (J.F.)

**Keywords:** microstereolithography, 3D printing, additive manufacturing, photo-curable polymer, micromanipulation, microtweezers, compliant mechanism

## Abstract

In this study, a three-dimensional (3D) micromanipulator mounted on a glass capillary is developed for handling biological samples, such as multicellular spheroids and embryos. To fabricate the micromanipulator, we developed an additive manufacturing system based on high-resolution microstereolithography using a 405-nm blue laser. The fabrication system makes it possible to fabricate 3D microstructures on a glass capillary with 2.5 µm lateral resolution and 25 µm layer thickness. We also demonstrated the capture and release of a spheroid with the micromanipulator fabricated using our additive manufacturing system. We showed that spheroids can be easily handled by a simple operation with minimal damage using a cage-like multiple finger structure. Additive manufacturing of tailor-made micromanipulators mounted on a glass capillary will be useful in biological and tissue engineering research.

## 1. Introduction

In recent years, research interests in tissue engineering and regenerative medicine, using induced pluripotent stem (iPS) cells and embryonic stem (ES) cells, have significantly increased [1,2,3,4]. Techniques for extracting the desired biological samples, such as cells, spheroids, and organoids from a culture dish and handling them are required, in order to conduct these studies more efficiently. Various techniques for handling biological samples of different sizes and morphologies have been proposed [5,6,7,8,9,10,11,12]. For instance, micropipette aspiration and separation of cells [7,8,9], cell manipulation using tweezers produced by micromachining and 3D printing [6,10,11,12], single cell handling using optical tweezers [13,14], and cell manipulation based on microfluidic systems [5,6,15,16] have been employed for various applications. Optical tweezers allow microscale samples to be manipulated remotely. However, they cannot extract samples from liquid into the air. Microfluidic techniques for manipulating cells and spheroids enable many samples to be handled simultaneously inside micro-channels, but such techniques are not suitable for the separation and transfer of cells in a culture dish. On the other hand, micropipettes are widely used as a technique for aspirating and extracting cells, embryos, and spheroids. However, in this method, cells are aspirated using a capillary with a smaller inner diameter than the diameter of spheroids, which may damage cells or produce mechanical stress [17]. On the other hand, microgrippers, made by micromachining, can be used to handle multiscale biological samples, such as DNA, single cells, and spheroids because they can be designed and produced according to target samples using high-precision microfabrication techniques [6,10]. Recently, tweezers utilizing 3D printed arms have been demonstrated [11,12]. However, simple two-dimensional end effectors are used in conventional micro grippers, hence challenges remain in terms of stable griping and extraction of samples. In addition, the conventional micromachined tweezers require high-precision control to drive the end effectors with high accuracy of position.

In this study, we propose a 3D-printed micromanipulation tool with multiple polymer microfingers that can easily handle biological samples, such as spheroids with a simple operation. A laboratory-scale high-resolution microstereolithography system was fabricated using a blue laser for additive manufacturing of the polymer microfingers. The manipulator utilizes the elastic deformation of microfingers, mounted on the tip of a glass microcapillary, to hold the spheroid. The spheroid can be easily held by pressing only the fingers against the bottom of the culture dish. When the captured spheroid is removed from the culture medium, it is trapped in a droplet of culture medium inside the microfingers. Air is subsequently delivered through the glass microcapillary to push out the spheroid into the culture medium and place it at the desired position. Through this approach, the proposed manipulator does not need to drive each finger using actuators, and can easily capture spheroids using only the elastic deformation of the fingers, resulting in reduction in cost, size, and weight of the manipulators. In addition, soft and fragile biological samples can be gripped with minimal damage as the target samples are not directly gripped with fingers. Therefore, the proposed method based on caging surrounded by multiple fingers offers simple and damage-free manipulation without the use of force sensors. Furthermore, additive manufacturing using microstereolithography enables the production of custom-made manipulators with different finger shapes and sizes based on a wide variety of biological samples.

## 2. Materials and Methods

### 2.1. High-Resolution Microstereolithography System Using a 405-nm Blue Laser

**Figure 1:**
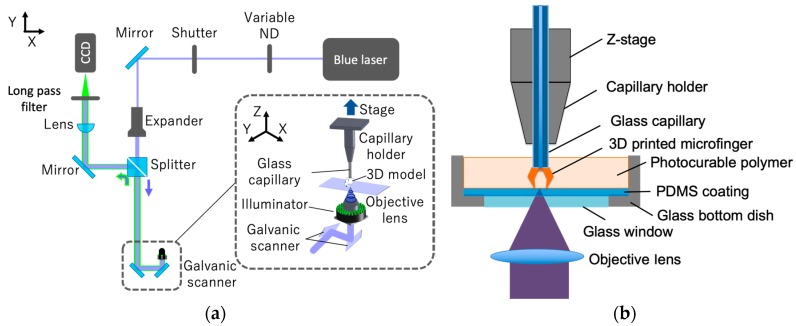
Microstereolithography system used to additively manufacture microfingers on top of a glass capillary: (**a**) Optical system; (**b**) Setup for fabricating microfingers at the end of a glass capillary.

We have previously developed several microstereolithography systems using femtosecond laser, blue laser, and ultraviolet laser capable of fabricating 3D models with high-resolution [18,19,20,21]. In this study, a holding jig was integrated into a top-down microstereolithography setup to hold the glass microcapillary using the 405-nm blue laser developed in our previous study [20]. Figure 1 shows an outline of the developed fabricating system. Laser light emitted from a blue semiconductor laser (Cobolt 06-MLD, Cobolt AB, Solna, Sweden, wavelength: 405 nm) is reflected using a galvanic mirror scanner (GM-1015, Canon Inc., Tokyo, Japan). The laser light was then introduced into an objective lens (PLN4X, Olympus Corp., Tokyo, Japan) with a numerical aperture of 0.1 and focused on the photo-curable polymer. A high aspect ratio structure with a height of 8.5 cm can be formed by raising and lowering the z stage on which the jig for mounting the glass capillary is installed. A top-down method was adopted in which the glass capillary was pulled up during a layer-by-layer process, while the focused laser spot was scanned in two-dimension at a high speed with galvanic mirrors. An observation system, consisting of a charge-coupled device (CCD) camera, a lens, and a green LED ring-shaped illuminator was used to determine the initial position of the end face of the capillary before additive fabrication. At the initial position, it was also confirmed that the blue laser beam was focused on the end face of the capillary to fix the first sliced layer of the microfingers stably. The initial setup using this observation system enables us to additively manufacture the microfingers on the microcapillary without harmful detachment and deviation. The laser power was adjusted using a variable neutral-density (ND) filter to optimize the fabrication condition. In addition, the scanning speed and scanning trajectory of the laser beam were optimized.

### 2.2. Surface Coating of Bottom Dish and Glass Capillary

In the microstereolithography system, the photo-curable polymer was stored in a glass bottom dish (diameter: 27 mm) for fabricating 3D models. The glass surface was coated with polydimethylsiloxane (PDMS) to prevent adhesion of the photo-curable polymer to the glass substrate at the bottom. A PDMS pre-polymer solution composed of silicone elastomer and a curing agent (10:1, Shin-Etsu Silicone, Tokyo, Japan) was coated on the glass bottom by spin coating at 2000 rpm for 10 s and then cured at 60 °C for 3 h.

### 2.3. Photocurable Polymer for High-Resolution Microstereolithography Using 405-nm Blue Laser

A photo-curable polymer suitable for high-resolution 3D microfabrication is indispensable for high-resolution microstereolithography using blue laser. Although, a photo-curable polymer suitable for UV light was used in our previous study [20], a novel photo-curable polymer that strongly absorbs blue light at a wavelength of 405 nm was developed in this study based on the photo-curable polymer proposed by LaFratta et al. [22] to reduce the linewidth and cure depth, in order to manufacture sophisticated microfingers with high resolution. The use of a microstereolithography system, with a blue laser and photo-curable polymer, ensures that micrometer resolution can be achieved without using the femtosecond pulsed laser, which is commonly used in two-photon microstereolithography systems [18,23].

The newly developed photo-curable polymer is a mixture of an acrylate resin (SR499, Sartomer Inc., Exton, PA, USA, 95.9 wt %) and a photopolymerization initiator (TPO, diphenyl (2,4,6-trimethylbenzoyl) phosphine oxide, Sigma-Aldrich, St. Louis, MO, USA, 1.0 wt %), polymerization inhibitor (2-tert-butyl-4-methylphenol, Sigma-Aldrich, St. Louis, MO, USA, 3.0 wt %), and blue light absorber (FDB-009, Yamada Chemical Co., Ltd., Kyoto, Japan, 0.1 wt %). We prepared the photocurable polymer by mixing the above ingredients with a mixer (ARE-250, Thinky Corp., Tokyo, Japan). In the process of mixing the materials, the mixture was first mixed in the mixing mode at 2000 rpm for 5 min and then degassed in the de-foaming mode at 2000 rpm for 5 min. Then, it was stirred for 24 h at 60 rpm in a ball mill. The mixed photopolymer can be used for at least 6 months.

The absorbance of the blue laser beam on the photo-curable polymer can be increased by mixing the blue light absorber, and the size of the cured voxel when the photo-curable polymer is irradiated with the blue laser beam can be reduced. After fabrication, the 3D models were washed with alcohol solvent (SOLFIT, 3-methoxy-3-methyl-1-butanol, Kuraray CO., Ltd., Tokyo, Japan) and ethanol for 10 min respectively.

### 2.4. Preparation of NIH/3T3 Spheroids

The capability of the microfingers to capture and release biological samples were evaluated using NIH/3T3 spheroids. NIH/3T3 cells were seeded in one well of a non-cell-adhesive round-bottom 96 well plate (Prime surface 96U, Sumitomo Bakelite, Tokyo, Japan) at a density of 3 × 103 cells/well and cultured for 1 day in Dulbecco’s modified Eagle’s medium (Sigma-Aldrich, St. Louis, MO, USA) supplemented with 10% fetal bovine serum (Sigma-Aldrich, St. Louis, MO, USA) and 1% penicillin-streptomycin (Thermo Fisher Scientific, Waltham, MA, USA) in a humidified environment at 37 °C/5% CO_2_. The formed NIH/3T3 spheroids were captured and released by using microfingers. The cytotoxicity, caused by manipulation of NIH/3T3 spheroid, was quantified by a lactate dehydrogenase (LDH) cytotoxicity detection kit (Takara, Shiga, Japan).

## 3. Results and Discussion

### 3.1. Resolution Evaluation of the Microstereolithography Using 405-nm Blue Laser

**Figure 2:**
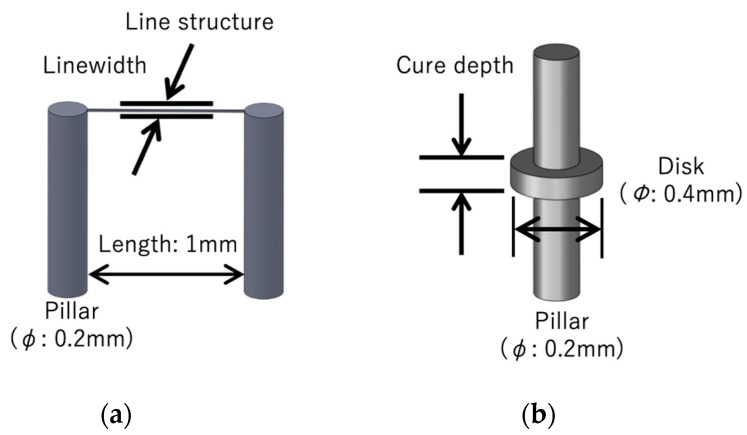
Test structures for the evaluation of the linewidth and cure depth: (**a**) Line structure supported by two pillars for measuring the linewidth; (**b**) overhanging disk structures for measuring the cure depth.

**Figure 3:**
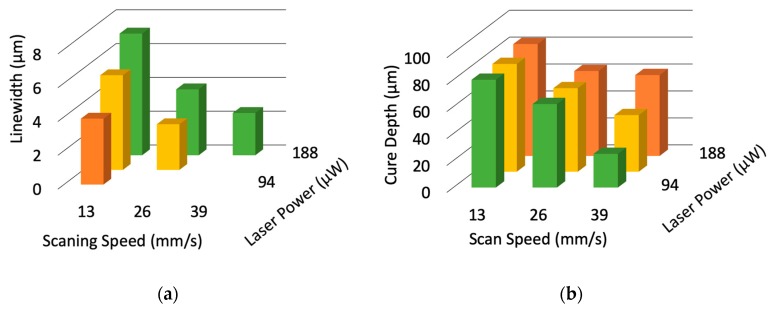
Dependence of width of the line structure and the cure depth of the disk on laser power and scanning speed: (**a**) Width of the line structure; (**b**) Cure depth of the overhanging disk.

**Figure 4:**
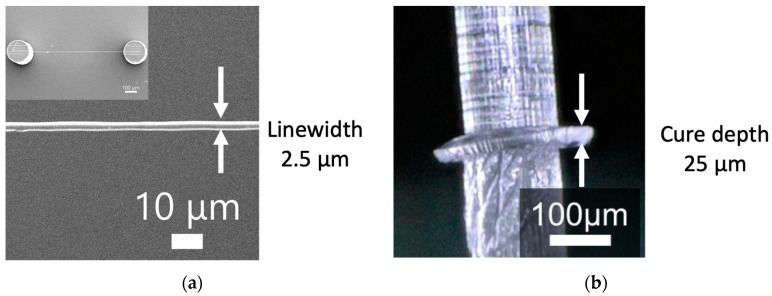
Minimum linewidth and cure depth: (**a**) Minimum width of the line structure; (**b**) minimum cure depth of the overhanging single-layer disk.

To evaluate the fabrication resolution of the laboratory-constructed microstereolithography system, we developed two types of test structures for measuring the lateral and depth resolutions. To measure the lateral resolution, we developed a line structure suspended with two pillars (Figure 2a). The line was developed by single scanning of the laser beam after fabricating the two supporting pillars made by piling up circular disks with a layer pitch of 10 μm. The widths of the line structures were measured by varying the laser power and scanning speed. We developed a pillar with an overhanging single-layer disk with a layer pitch of 10 μm to measure the depth resolution (Figure 2b). The thicknesses of the overhanging disks were also measured by varying the laser power and scanning speed. Figure 3a,b show the dependence of the widths of the line structures and cure depths of the disks on laser power and scanning speed. Both the linewidth and the cure depth decreased as the scanning speed increased and the laser power decreased. Figure 4a,b show the structures of the minimum linewidth and minimum thickness, respectively. The minimum linewidth of 2.5 μm was obtained at a laser power of 188 μW and a scanning speed of 39 mm/s. The suspended line was straight without any distortion after washing out the uncured polymer. The minimum cure depth of 25 μm was obtained at a laser power of 94 μW and a scanning speed of 39 mm/s. These parameters are useful for making 3D structures with high precision.

### 3.2. Fabrication of a Micromanipularor for Handling a Spheroid

**Figure 5:**
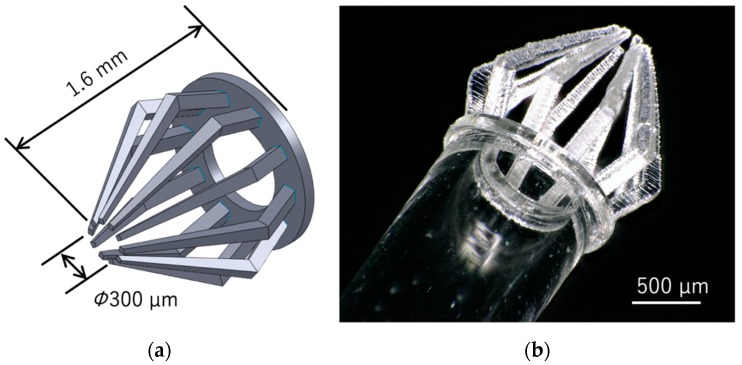
Microfingers mounted on a glass capillary: (**a**) 3D CAD model; (**b**) Bird’s eye view.

A manipulator with 10 microfingers at the end face of a glass capillary was designed as a micromanipulator for grasping spheroids in a culture dish filled with a culture medium without serious damage (Figure 5a). The inner diameter and outer diameter of the glass capillary are 920 µm and 1390 µm, respectively. The inlet diameter of the gathered cage-like fingertip enclosure is 300 µm, and the height of the finger is 1.6 mm. Since the microfingers are made of acrylate polymer, the tip is deformed when the finger is pressed against the glass substrate, and the fingers are closed. This feature was utilized to encapsulate the spheroid in the cage surrounded by microfingers. Although, the diameter of the inlet of the cage is slightly smaller than that of a spheroid, spheroids are sufficiently soft to deform without damage and pass through the inlet of the cage when the fingers are pressed. Figure 5b shows photographs of the microfingers fabricated on the end face of the glass capillary using the microstereolithography system with a blue laser. The laser power, laser scanning speed, hatching distance, and layer pitch were 47 µW, 2.6 mm/s, 3 µm, and 20 µm, respectively. The fabrication time was 36 min.

### 3.3. Analysis of the Mechanical Properties of Microfingers

**Figure 6:**
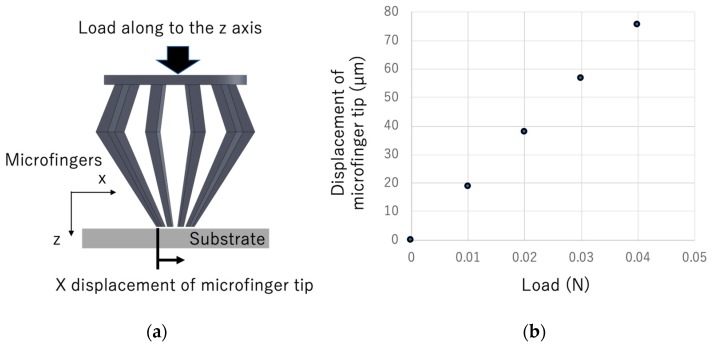
Analysis of x displacement of the microfinger under load: (**a**) Simulation model; (**b**) Displacement of the microfinger along the x axis.

**Figure 7:**
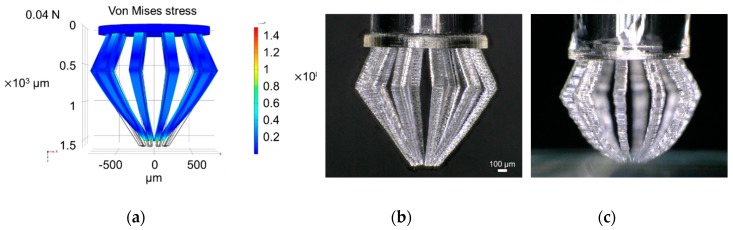
Bending of the microfinger on the substrate: (**a**) Simulation results under a load of 0.04 N; (**b**,**c**) demonstration of microfingers mounted on a glass capillary before and after pressing them on the substrate.

The von Mises stress and deformation when the designed microfingers were pressed against a substrate were calculated using finite element software (COMSOL multiphysics version 5.4, COMSOL, Inc., Palo Alto, CA, USA), in order to confirm the gripping operation of the microfingers. In the analysis, the Young’s modulus and Poisson’s ratio of the microfingers was assumed to be 2.5, and 0.4 GPa, respectively. The coefficient of friction between the fingertip and the substrate was assumed to be 0.4. Figure 6a illustrates the simulation model of microfingers pressed against the substrate. Figure 6b shows the calculation results of the displacement of the fingertip in the x direction when a load is applied in the z direction. Figure 7a shows a calculation example for the stress and deformation when a load of 0.04 N is applied. It can be observed from the figure that, by pressing the microfingers against the substrate, each tip is bent and the microfingers can be gathered and closed.

Figure 7b,c show photographs before and after pressing the microfingers on the substrate. These results demonstrate that the tips of the microfingers are distorted inward when the microfingers are pressed against the substrate, which agree with simulation results. The force required to deform the microfingers was confirmed to be 0.03 N by measuring with a force sensor (Compression Load Cell, LMA-A-5N, Kyowa Electronic Instruments Co., Ltd., Tokyo, Japan). This value is almost consistent with the simulation results using COMSOL multiphysics.

### 3.4. Manipulation of the Spheroid Using the Micromanipulator

**Figure 8:**
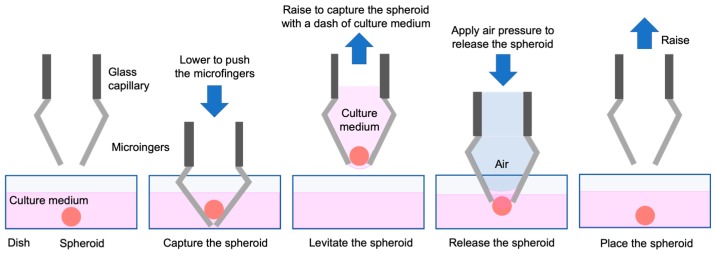
Handling procedure of a spheroid using microfingers mounted on a glass capillary.

Figure 8 shows how to pick up and release a spheroid in a culture dish using the developed micromanipulator. First, the center of the microfingers, which corresponds to the position of the spheroid, is aligned; the microfingers are pressed against the dish, and are deformed to capture the spheroid. The spheroid is slightly deformed and enters the cage comprising multiple microfingers. When the microfingers are lifted from the culture medium, the spheroid is included in a droplet of culture medium and removed from the culture dish. When the captured spheroid is placed at the desired position, air is pushed out from the glass capillary to discharge the spheroid together with the culture medium into a dish. This demonstrates that the spheroid can be placed at the desired position.

To verify the validity of our micromanipulator, we constructed a manual manipulation system for handling spheroids using a microscope (VHX-5000, Keyence Corp., Osaka, Japan) with an objective lens (VH-Z25 (magnification: 25–175), Keyence Corp., Osaka, Japan). In this system, the manipulator is mounted on XYZ stages and tilt stages, so it can be manually operated accurately, while observing the tip of the microfingers with the microscope. To extract the captured spheroid, we connected a small rubber pipettor to the other end of a glass capillary. In experiments, air is pushed out by manually pushing the rubber pipette to extract the spheroid from microfingers.

**Figure 9:**
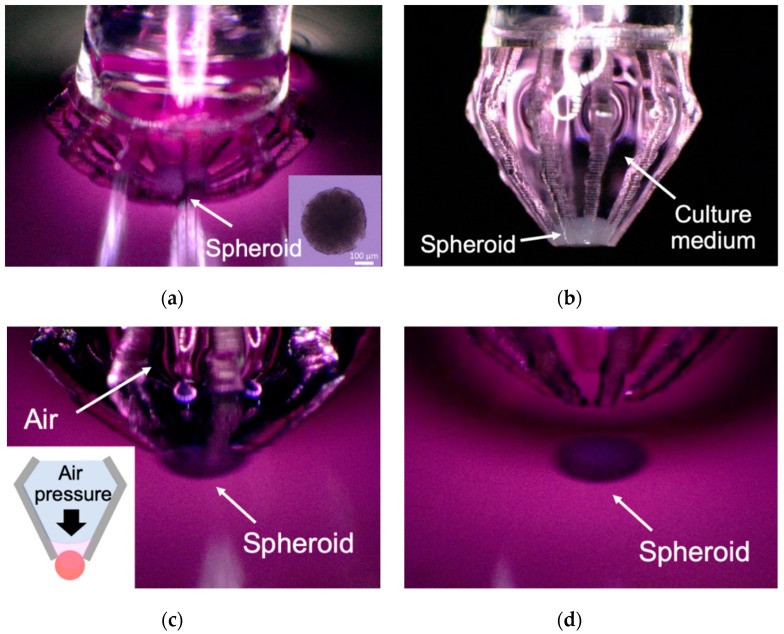
Demonstration of handling of a spheroid using microfingers mounted on a glass capillary: (**a**) Capturing the spheroid; (**b**) grasping the spheroid encapsulated in a culture medium; (**c**) Releasing the spheroid in a culture medium by applying air pressure; (**d**) placing the spheroid at the bottom of a dish.

Figure 9 shows the capture and release of a spheroid by the micromanipulator. The spheroid can be grasped, while pulling the microfingers out of the culture medium and retaining the culture medium inside the cage, surrounded by microfingers (Figure 9b). When the spheroid is held in the cage, it is not exposed to air because it is surrounded by the culture medium; thus, damage to the spheroid is minimal. To insert the spheroid into the culture medium again, air pressure was slightly applied and the spheroid was pushed out from the capillary (Figure 9c). At this time, air does not leak from the side gap of the microfingers, which is due to the effect of surface tension of the culture medium, and the culture medium is pushed out only from the tip of the microfingers, while enclosing the spheroid. Therefore, the spheroid can be ejected gently by air pressure such that physical damage to the spheroid is minimal. Finally, the spheroid is placed at the desired location on the bottom of the dish.

**Figure 10:**
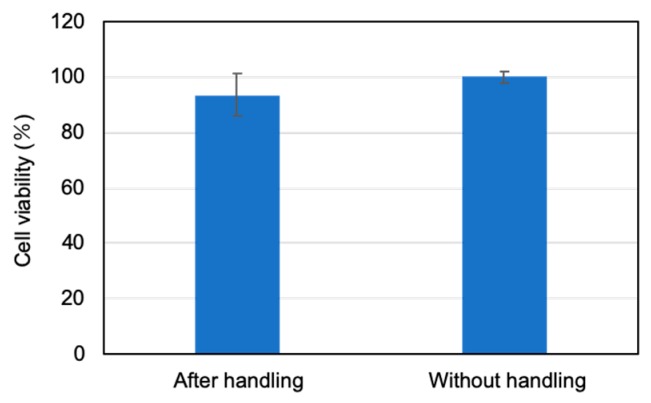
Experimental results of lactate dehydrogenase (LDH) cytotoxicity evaluation.

The damage to the spheroid after the capturing and releasing experiments was evaluated. The lactate dehydrogenase (LDH) activity evaluation method [24] was used for the evaluation experiment. This method quantifies intracellular substances when the cell wall is broken, and can evaluate the cytotoxicity. The result of the evaluation experiment shows that there is no significant difference between the cells after capturing and releasing by the micromanipulator and the cells without manipulation (Figure 10). It was confirmed that following the manipulation experiments the spheroids can be cultured without serious damage. 

## 4. Conclusions

In this study, we established a method to additively fabricate a micro-3D part to the end-face of a glass microcapillary, using a high resolution microstereolithography system with a blue laser. To demonstrate its application, we developed a micromanipulator to capture and release spheroids in a culture dish. In this manipulator, a spheroid can be captured inside a cage, comprising multiple microfingers with a droplet of culture medium supported by the microfingers. The spheroid can be easily dispensed to the culture medium by applying air pressure through the microcapillary. Therefore, the micromanipulator offers a simple and damage-free handling of spheroids. Furthermore, these 3D-printed micromanipulators will be useful for more versatile handling of biological samples, such as cells, spheroids, and embryos in biological research and regenerative medicine, given the size and shape of the microfingers can be easily customized and produced using microstereolithography.
